# An ethical framework for cardiac report cards: a qualitative study

**DOI:** 10.1186/1472-6939-6-3

**Published:** 2005-03-28

**Authors:** Shawn A Richard, Shail Rawal, Douglas K Martin

**Affiliations:** 1 University of Toronto Joint Centre for Bioethics, 88 College Street, Toronto, Ontario, M5G, Canada 1L4; 2 Department of Health Policy, Management and Evaluation, McMurrich Building, 2nd Floor, 12 Queen's Park Crescent West, Toronto, Ontario, M5S 1A8, Canada

## Abstract

**Background:**

The recent proliferation of health care report cards, especially in cardiac care, has occurred in the absence of an ethical framework to guide in their development and implementation. An ethical framework is a consistent and comprehensive theoretical foundation in ethics, and is formed by integrating ethical theories, relevant literature, and other critical information (such as the views of stakeholders). An ethical framework in the context of cardiac care provides guidance for developing cardiac report cards (CRCs) that are relevant and legitimate to all stakeholders. The purpose of this study is to develop an ethical framework for CRCs.

**Methods:**

Delphi technique – 13 panelists: 2 administrators, 2 cardiac nurses, 5 cardiac patients, 2 cardiologists, 1 member of the media, and 1 outcomes researcher. Panelists' views regarding the ethics of CRCs were analyzed and organized into themes.

**Results:**

We have organized panelists' views into ten principles that emerged from the data: 1) improving quality of care, 2) informed understanding, 3) public accountability, 4) transparency, 5) equity, 6) access to information 7) quality of information, 8) multi-stakeholder collaboration, 9) legitimacy, and 10) evaluation and continuous quality improvement.

**Conclusion:**

We have developed a framework to guide the development and dissemination of CRCs. This ethical framework can provide necessary guidance for those generating CRCs and may help them avoid a number of difficult issues associated with existing ones.

## Background

A growing number of health care report cards are now available, and they are increasingly being used to increase the profiles of high performing hospitals amongst consumers [1-3]. However, the recent proliferation of health care report cards has occurred in the absence of an ethical framework to guide their development and implementation. An ethical framework is a consistent and comprehensive theoretical foundation in ethics, and is formed by integrating ethical theories, relevant literature, and other critical information, such as the views of stakeholders. It provides guidance for developing new practices and for challenging and evaluating existing ones. Gormley and Weimer[4] developed a normative framework for organizational report cards through their own expertise as policy analysts, and input from scholars and individuals involved in the design and implementation of organizational report cards. However it was not derived from ethical theory or grounded in the systematically described views of stakeholders.

Health care report cards are most well developed in cardiac care. An ethical framework would provide necessary guidance for those generating cardiac report cards (CRCs) and may help them avoid a number of difficult issues associated with existing report cards. 'Gaming' and uncertainty about the quality of report card data have been cited as impediments to reliable outcomes measures and a reason to limit the public release of report card data [5,6]. Uncertainty also exists as to whether report cards have empowered patients and/or improved health care quality[7-10]. Other ethical and practical issues, such as balancing the public's desire for provider-specific outcomes measures with cardiac care providers' desires to limit the amount and type of information released to the public, affect the content and legitimacy of CRCs.

An ethical framework can identify points of ethical concern for practitioners, patients, policy makers and researchers. And it can aid in the development, implementation and improvement of future generations of CRCs.

The purpose of this study is to develop an ethical framework for CRCs.

## Methods

### Design

Forming this ethical framework has been a three step process. First, we analyzed the relevant ethical issues in an earlier article [11]. A summary of these issues is presented in table 1. Next we described stakeholders' views in two previous papers [12] (a paper on patients' views has been submitted for publication). Finally, this study used a Delphi method with a panel of stakeholders to synthesize these insights into an ethical framework.

**Table 1:** Summary of the ethical issues concerning cardiac report cards (adapted from Nast S, Richard SA, Martin DK. Ethical issues related to cardiac report cards. *Can J Cardiol*. Mar 1 2004;20(3):325–328.)

**Ethical issue**	**Description**
Quality	•Quality operationalizes the ethical principles of beneficence and non-maleficence
	•Report cards may improve quality of care through external pressure from an informed public
	•Report cards may impede improvements to quality by generating anger and defensiveness

Informed Consent	•Informed consent operationalizes the ethical principle of autonomy
	•To make health care decisions, patients need and want information about their medical options
	•Report cards have the potential to provide this information and thus facilitate informed consent

Equity	•Equity operationalizes the ethical principle of justice
	•Health equity between regions is an important consideration in publicly funded health systems
	•Report cards must address policy makers to affect regional inequities in health care

Legitimacy	•Legitimacy operationalizes the ethical principle of justice, in this case deliberative forms of democratic justice
	•Report card authors must ensure that report cards are and perceived to be legitimate
	•The legitimacy of report cards will depend on their ability to meet stakeholders' reasonable expectations

The Delphi method allows a panel of stakeholders to generate ideas on a given topic and to reach a consensus on the relative importance of those ideas [13,14]. This study used a three-round modified Delphi method to identify and reach agreement on the elements of an ethical framework for CRCs.

### Participants and sampling

Participants were selected from two previous studies conducted by this research team. The first study described the views of cardiac care administrators, cardiac surgeons, cardiac nurses, cardiac patients, cardiologists, members of the media, and outcomes researchers about CRCs [12]. The second study described the views of cardiac patients (a paper on this study has been submitted for publication).

We selected participants for our Delphi panel so that the views and interests of each stakeholder group were represented. Our final panel consisted of 13 panelists: 5 cardiac patients, 2 administrators, 2 cardiac nurses, 2 cardiologists, 1 member of the media, and 1 outcomes researcher. We included a critical mass of patients in order to balance potential or perceived power differentials and enhance the comfort level of participating patients.

### Data collection and analysis

The Delphi process consisted of three rounds. Round 1 was conducted using electronic communication. We provided three papers based on previous research to each panelist in order to provide background information on the topic. The first paper, *Ethical issues related to cardiac report cards*, identifies ethical issues related to CRCs, and provided panelists with an ethical analysis [11]. The other two papers, *Stakeholders' views about cardiac report cards *[12] and *Patients' views about cardiac report cards*, describe stakeholders' views relating to CRCs (a paper on patients' views has been submitted for publication). We asked the panelists to identify key issues that emerged from the papers, and to suggest other issues that may have been missing but were relevant to the ethics of CRCs. We synthesized the feedback from the panelists and organized the items into categories that originated from the panelists' feedback. We combined the items into a draft ethical framework, using the ethical language of the panelists.

Round 2 was conducted as a face-to-face round-table discussion. First, we disseminated the draft ethical framework developed in Round 1 to the panelists by mail and email. Then the panelists came together for a discussion of key issues from the draft ethical framework. The discussion was facilitated by a member of the research team who encouraged panelists to debate the issues amongst themselves and develop a consensus on the key items. Two other members of the research team independently recorded the round table discussion. We used the data gathered at the meeting to refine the framework's key items. After we organized the feedback into their corresponding categories, we integrated new comments into the existing draft framework and examined it to identify any inconsistencies. At the end of Round 2, we had a refined draft ethical framework.

Round 3 was conducted electronically, we disseminated the new draft ethical framework by mail and email to the panelists for final refinements. We organized the panelists' feedback according to the refined list of categories from Round 2. We integrated new comments into a refined, ethical framework and re-examined it for inconsistencies. The result of Round 3 was a final ethical framework for CRCs.

### Research ethics

This study was approved by the Committee on Research with Human Subjects at the University of Toronto. We obtained informed consent from each panelist and kept all data confidential and anonymous to those who were not directly involved in the project.

## Results

**Figure 1:**
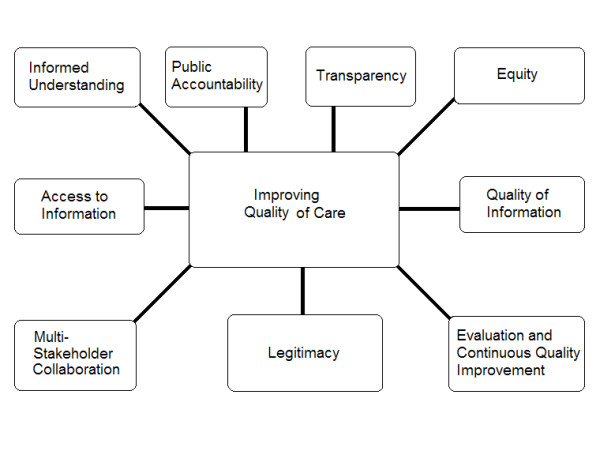
Ten principles for cardiac report cards.

Figure 1 provides an overview of the ethical framework. In this section we will describe the items that form the framework and discuss the interrelationships between items. Although the panelists decided that improving the quality of cardiac care was the overriding principle in this ethical framework, they also decided that the principles should not be ranked because they are interrelated, not independent.

### Improving quality of care

Improving the quality of cardiac care should be the fundamental objective driving the development and implementation of CRCs. In meeting this objective, report cards should also maximize the public good by ensuring equitable quality of care within and between regions. Providing stakeholders with information on the quality of cardiac care may be a necessary and effective impetus for improving the quality of cardiac care. All of the other principles described below should contribute to the overall goal of improving the quality of cardiac care.

### Informed understanding

CRCs should help inform patients about the quality of cardiac care provided within regions, by healthcare organizations, teams and individual health providers. They should provide information that users identify as relevant to their needs and wants. In particular, the information contained within CRCs must be available and comprehensible to the public – i.e. outcome measures should be provided with an adequate amount of relevant context to interpret data. Providing patients with information is a useful end in itself, even if that information is not utilized by patients in making informed choices. For example, a patient can derive comfort from knowing that his/her health care provider meets an acceptable standard of cardiac care. Report cards should contain physician specific qualitative information, such as a description of patient experiences. Valid and reliable qualitative measures as to the quality of cardiac care will have to be developed. However, physician-specific quantitative measures should not be reported within report cards unless data meet reliable criteria, such as originating from a sufficiently large sample size over a sufficient period of time, utilizing commonly accepted risk-adjustment methods, and including a validation process. Further individual physicians should not be ranked based on the outcomes of these measures. Rather physicians should be identified as meeting or not meeting an acceptable standard of care – which will have to be defined.

### Access to information

Information that is collected for and presented in CRCs must remain public property, and access to this information must be free. Public ownership of report cards may prevent them from perpetuating disparities in access to information. Panelists saw public ownership of report cards as a way of engaging the public and other relevant stakeholders in improving the quality of cardiac care. There should be two types of report cards: one for health care professionals and policy makers, and one for patients and the public. There are a number of reasons why this is necessary. The two audiences have different interests, which necessitates different content, and different levels of understanding, which necessitates different formats. However both types of report cards should remain public property. Those responsible for CRCs should use multiple dissemination methods that will reach all stakeholders. These methods should include physician-patient discussions, open forums and discussion panels.

### Equity

CRCs should address issues of equity. They should include information on the quality of cardiac care and allocation of resources in different health care institutions and geographic regions. Equity interplays with the principle of access to information. Ineffective dissemination methods may exacerbate existing disparities amongst cardiac patients. Living in rural areas or having limited access to computers and the internet should not prevent patients from having access to CRCs. The principle of equity insists that all cardiac patients have access to the information contained within CRCs.

### Transparency

CRCs should be available to the public because they enhance transparency regarding how the cardiac care system functions. By noting strengths and deficiencies within the cardiac care system, CRCs will enable health care professionals and health policy makers to make necessary changes to enhance the quality of cardiac care, and will enhance public accountability for quality cardiac care. Transparency is also necessary for patients to achieve an informed understanding of the quality of cardiac care, by ensuring consistent access to relevant and accurate information. In this way the principles of transparency, informed understanding, access to information, and quality of information are interrelated. Thus transparency is instrumental because it facilitates other desired outcomes.

### Public accountability

Public accountability refers to the obligation on the part of health care professionals to accept responsibility for the quality of care they provide. Public accountability can be operationalized in two ways: (1) accountability to the public independent of public participation; and (2) accountability to the public enforced through a partnership with patients. CRCs can help signify health care providers' willingness to be accountable for the quality of care they provide and satisfy the public's need for accountability. Transparency and access to information are key aspects of public accountability.

### Multi-stakeholder collaboration

Report cards should be developed in collaboration with all stakeholders, including patients. Those who develop and implement CRCs must involve stakeholders early and throughout the process of report card development, and balance the needs of different stakeholders. In their development and implementation, CRCs can foster collaboration and reduce anger and defensiveness. Multi-stakeholder collaboration and transparency are a means of ensuring that CRCs are viewed as legitimate to all relevant stakeholders.

### Legitimacy

Legitimacy, the moral authority of CRCs, must be a key aspect of the development and implementation of CRCs. Patients' views on what is needed in CRCs are necessary to support the principle of legitimacy because patients are at the heart of cardiac care. Patients' views are inherently important, and outcome measures that address patients' wants, such as patient experience measures, should be included in report cards. Traditional quality indicators of morbidity and mortality will be insufficient to meet these needs. Like morbidity and mortality, patient experience measures must also be of high quality, realizing the principle of quality of information. The legitimacy of report cards is also derived from transparency and public ownership of the information contained within them.

### Quality of information

Report card data must be of high quality. This means that data must be without bias and risk-adjusted and should also be produced and disseminated by an independent and objective third party. Report cards should provide stakeholders with information on matters that can be addressed by the individuals, groups, or organizations which are being evaluated. Similar institutions should be compared to each other – for example, an urban teaching hospital should be compared to a similar urban teaching hospital. This sensitivity to the similarity of institutions should also encompass differences in patients' geographical proximity to institutions. In addition, CRCs must protect patient privacy. The quality of report card data and access to that data are important if report cards are to help initiate changes for improvement in practice sites, or be tied to government funding.

### Evaluation and continuous quality improvement (of reporting)

CRCs should be useful to stakeholders. Continuous quality improvement and effectiveness monitoring initiatives should be in place to ensure report cards meet their intended goals. The measures contained within CRCs should be constantly reviewed, through multi-stakeholder collaborations, to ensure that they provide a fair assessment of the quality of care being evaluated, which requires transparency of the information gathered. Access to report card data by individuals from each stakeholder group is necessary for continuous quality improvement to be achieved.

## Discussion

This study developed an ethical framework to guide the development and implementation of CRCs (see Figure 1). To our knowledge, this is the first ethical framework developed for CRCs, or for health care report cards in general. Gormley and Weimer developed a normative framework for report cards that helped identify some key issues [4]. However, its impact was limited because it was not explicitly grounded in moral theory or the systematically described views of stakeholders. Our framework is an advance because it is grounded in ethical theory and in the systematically described views of stakeholders, and thus can provide guidance and is applicable in real life policy and practice.

Improving the quality of cardiac care is the primary ethical objective of CRCs. There was consensus on this across all stakeholder groups. Thus, efforts to develop and disseminate a CRC ought to be congruent with this fundamental objective. The panelists identified nine other elements: access to information, informed understanding, equity, transparency, public accountability, multi-stakeholder collaboration, legitimacy, quality of information, and the evaluation and continuous quality improvement of reporting.

This ethical framework for CRCs can be used to facilitate the development of report cards. It can provide guidance for addressing difficult issues associated with existing report cards. For example, report cards have been met with anger and defensiveness from clinicians. In response, this framework suggests that clinicians and other stakeholders be involved throughout the process of report card development, giving them opportunities to identify their concerns.

The public release of quality of care data has been met with considerable controversy, particularly in the United States [15]. Studies suggest that clinicians are skeptical of data contained in report cards and that such data has little impact on referral decisions [8]. Further, cardiac patients have made limited use of previous CRCs to inform decision-making on their care [16,17]. Questions about the legitimacy of earlier CRCs may be one source of these issues. This framework provides guidance for enhancing the scientific, political and moral legitimacy of report card data. For example, for report cards to be more scientifically and morally legitimate to clinicians, physician-specific quantitative measures should not be reported within report cards unless data meet reliable criteria, such as originating from a sufficiently large sample size over a sufficient period of time, utilizing commonly accepted risk-adjustment methods, and including a validation process. These methods will help ensure the subjects of CRCs are evaluated fairly. And for report cards to be legitimate to patients, outcome measures addressing their information needs must be included, and patients must be involved in the development and implementation of CRCs. The success of qualitative measures that are currently being employed in other areas of health care suggests such measures are possible [18].

The framework suggests that individual providers should be identified as meeting or not meeting an acceptable standard of care, and that physician-specific qualitative information be provided to assist cardiac patients in developing an informed understanding of their care. This view is congruent with findings that patients value information on the interpersonal aspects of care, such as communication and timeliness [19]. Since the framework suggests that clinicians be reported as meeting an acceptable or unacceptable standard of care, and not ranked individually, future report cards should significantly curb some of the incentives for 'gaming' [5]. In addition, this ethical framework recommends that report card authors involve health care providers in the development of outcomes measures and implement continuous quality reviews of outcomes measures to ensure that they provide a fair assessment of the quality of care being evaluated.

The framework suggests that information contained in report cards must remain publicly owned, that the public ought to have access to this information without charge, and that such information remain in the public domain. Public ownership of reports cards was seen as a method of ensuring that report cards do not perpetuate disparities in access to information. It was further viewed as a means of engaging the public and other relevant stakeholders in the process of improving quality of care. In that sense, public ownership can be seen as one means of facilitating public accountability.

The items identified in this ethical framework are interrelated. Transparency enhances public accountability by enabling health care professionals and policy makers to identify and correct deficiencies in the quality of cardiac care. Moreover, it is required if patients are to achieve an informed understanding of the quality of cardiac care. Transparency also plays an important role in the legitimacy of CRCs. Together multi-stakeholder collaboration and transparency are a means of securing the legitimacy of CRCs amongst stakeholders. The legitimacy of report cards is also derived from public ownership of the information contained within them. In this ethical framework, the quality of and access to report card data are important if report cards are to help initiate changes for improvement in practice sites, or be tied to government funding. A CRC both describing and comparing the availability of health care to citizens by region can serve, in part, as an indicator of how fairly health care resources are being allocated. Thus CRCs should include information on the quality of cardiac care and allocation of resources in different health care institutions and geographic regions. They are also necessary for continuous quality improvement of report card data.

The primary strengths of this study are that it is grounded in ethical theory and in the experiences and views of relevant stakeholders in cardiac care. Our Delphi panel consisted of a diverse range of participants from cardiologists, to cardiac patients, to members of the media. The diversity of our panel lends confidence to the validity of our findings [13]. Although a substantial body of literature on health care report cards exists, our framework is the first to provide ethical guidance on the development and dissemination of such reports. Further, it is the first to be constructed through multi-stakeholder collaboration.

### Limitations

The primary limitation of this research is its generalizability. Our framework reflects the views of the thirteen panelists who participated in our Delphi rounds, and may not be generalizable to other contexts. However, many of the items in our framework are congruent with the work of Marshall, Romano, and Davies who described strategies to maximize the impact of health care report cards [20], and with Gormley and Weimer's normative framework for organizational report cards [4]. This would suggest that our framework might be applicable in other contexts and to other types of health care report cards.

## Conclusion

We have developed an ethical framework to guide the development, implementation and improvement of CRCs. CRCs ought to improve the quality of cardiac care and should be informed by the following items: access to information, informed understanding, equity, transparency, public accountability, multi-stakeholder collaboration, legitimacy, quality of information, and the evaluation and continuous quality improvement of reporting.

## Competing interests

The author(s) declare that they have no competing interests.

## Authors' contributions

DKM conceived the study and participated in the data analysis and writing the paper. SAR had primary responsibility for data collection and analysis, and writing the paper. SR participated in data collection and analysis, and writing.

## Pre-publication history

The pre-publication history for this paper can be accessed here:


